# The Trajectory of Antibody Responses One Year Following SARS-CoV-2 Infection among Indigenous Individuals in the Southwest United States

**DOI:** 10.3390/v16101573

**Published:** 2024-10-05

**Authors:** Claire P. Smith, Rachel M. Hartman, Alexa M. Kugler, Verlena Little, Owen R. Baker, Tarayn A. Fairlie, Reinaldo E. Fernandez, Melissa B. Hagen, Elvira Honie, Oliver Laeyendecker, Claire M. Midgley, Dennie Parker, Marqia Sandoval, Saki Takahashi, Laura L. Hammitt, Catherine G. Sutcliffe

**Affiliations:** 1 Department of Epidemiology, Johns Hopkins Bloomberg School of Public Health, Baltimore, MD 21205, USA; 2 Department of International Health, Johns Hopkins Bloomberg School of Public Health, Baltimore, MD 21205, USA; 3 Department of Medicine, Division of Infectious Diseases, Johns Hopkins School of Medicine, Baltimore, MD 21205, USA; 4 Division of Coronaviruses and Other Respiratory Viruses, National Center for Immunization and Respiratory Diseases, Centers for Disease Control and Prevention, Atlanta, GA 30333, USA; 5 Division of Intramural Research, National Institute of Allergy and Infectious Diseases, National Institutes of Health, Bethesda, MD 20892, USA

**Keywords:** SARS-CoV-2, antibody response, Indigenous health

## Abstract

SARS-CoV-2 antibody kinetics based on immunologic history is not fully understood. We analyzed anti-spike and anti-nucleocapsid antibody responses following acute infection in a cohort of Indigenous persons. The models of peak concentrations and decay rates estimated that one year after infection, participants would serorevert for anti-nucleocapsid antibodies and remain seropositive for anti-spike antibodies. The peak anti-spike concentrations were higher for individuals vaccinated prior to infection, but the decay rates were similar across immunologic status groups. Children had significantly lower peak anti-spike concentrations than adults. This study affirms the importance of continued vaccination to maintain high levels of immunity in the face of waning immunity.

## 1. Introduction

In the four years since SARS-CoV-2 first emerged, evaluating immune responses to infection has become increasingly challenging in the face of complex immunologic exposure histories. As of March 2024, over 774 million cases and over 7 million deaths have been officially reported to the World Health Organization, and over 13.5 billion doses of SARS-CoV-2 vaccines have been administered globally [1]. As a result, most individuals have now acquired immunity through infection or vaccination, with many individuals having been both infected and vaccinated [2].

Infection or vaccination produces a strong humoral immune response in most individuals, with increases in anti-spike (S) protein and anti-receptor binding domain (RBD) IgG antibody levels, which are correlated with neutralizing activity [3]. A decline in antibody levels has been observed over time, with estimates of a half-life ranging from 85 to over 300 days [4,5,6,7]. Among vaccinated individuals with no previous SARS-CoV-2 infection, considerable waning of antibody levels has been observed by 4 to 6 months following the last dose of the vaccine [8,9]. Individuals with hybrid immunity (including those vaccinated after infection and those infected after vaccination) have documented greater breadth and magnitude of antibody response than those vaccinated or infected alone [3,10,11]. After infection, anti-nucleocapsid (N) antibodies are produced and then also decay steadily, with increases being observed following reinfection [3,11]. Age, sex, and the severity of acute illness have been observed to affect the rate of antibody decay among adults, with greater initial IgG responses being observed for hospitalized individuals, older adults, and males. Females and younger adults demonstrated more stable antibody levels over time [4,6,8].

In the United States, Indigenous populations have experienced a disproportionate burden from COVID-19, with higher rates of infection, hospitalization, and mortality than non-Hispanic White Americans [12]. As a result, Indigenous communities mounted successful vaccination campaigns and had the highest vaccine coverage of any racial/ethnic group after introduction [13]. Despite this, Indigenous persons are underrepresented in COVID-19 research, including studies evaluating the immune response to infection [14,15]. To address this gap, here, we describe the anti-S and anti-N antibody peak levels and decay rates and associated factors 12 months following acute SARS-CoV-2 infection among a cohort of Indigenous persons.

## 2. Materials and Methods

### 2.1. Study Population and Procedures

This cohort study was conducted in two rural communities in Navajo Nation (Chinle, AZ and Tuba City, AZ, USA) and in Whiteriver, AZ in the White Mountain Tribal Lands. The methods have been previously described [16]. Briefly, outpatients and inpatients testing positive for SARS-CoV-2 at participating health facilities were recruited for enrollment from February 2021 to August 2022. Written informed consent was obtained from adult (≥18 years) participants, and parental permission was obtained for participants aged <18 years. Written assent was also obtained from minor participants aged 7 to 17 years.

Participants had a baseline visit and follow-up visits at 1, 3, 6, and 12 months. At each visit, participants completed a questionnaire, and a blood sample was collected. The baseline questionnaire collected information on demographics, the acute illness, prior receipt of COVID-19 vaccines, and prior SARS-CoV-2 infections. The follow-up questionnaires collected information on current symptoms and measures of physical and mental health. At baseline and 3, 6, and 12 months, a chart review was completed. The baseline chart review collected information on underlying medical conditions, symptoms of and treatment for the acute illness, and prior receipt of COVID-19 vaccines. Follow-up chart reviews collected information on symptoms, COVID-19 vaccines received, and SARS-CoV-2 test results in the time since the last visit. State immunization information systems were also reviewed to confirm COVID-19 vaccines received.

Blood samples were processed and stored as serum at −80 °C until testing. Specimens were sent to Johns Hopkins University for testing using the Meso Scale Discovery (MSD) V-PLEX SARS-CoV-2 Panel 2 (IgG) KitTM (original Wuhan strain; Mesoscale Diagnostics LLC, Rockville, MD, USA), which is an electrochemiluminescence immunoassay, following the manufacturer’s protocol [17]. Results were converted to the World Health Organization International Standards binding antibody units (BAUs) [18] using conversion factors provided by the manufacturer and interpreted as seropositive or seronegative using the recommended signal thresholds.

### 2.2. Key Definitions

Immunologic exposure status at baseline was defined based on COVID-19 vaccination and history of infection prior to the acute SARS-CoV-2 infection at baseline. Vaccination status was defined based on chart review as unvaccinated (PreVax−) or having received any dose of an approved COVID-19 vaccine at least 14 days prior to the current infection (PreVax+). History of infection was defined, based on self-report or anti-N concentration at baseline (restricted to samples collected within one week of illness onset of the current infection), as having a history of infection (PreInf+; either self-reported prior infection or seropositive for anti-N) or not (PreInf−). Using this information, four immunologic exposure groups were defined: (1) history of vaccination but not infection (PreVax+/PreInf−); (2) history of infection but not vaccination (PreVax−/PreInf+); (3) history of vaccination and infection (PreVax+/PreInf+); and (4) history of neither vaccination nor infection (PreVax−/PreInf−).

Presence of comorbidities was defined based on chart review as underlying medical conditions associated with an increased risk of severe COVID-19 disease [19], including cancer, chronic kidney disease, chronic liver disease, chronic lung disease, COPD, asthma, cystic fibrosis, dementia or other neurologic conditions, diabetes, disabilities, heart conditions, hypertension, HIV, immunocompromisation, mental health conditions, overweight and obesity, pregnancy, sickle cell disease, thalassemia, being a current or former smoker, receipt of organ or stem cell transplant, stroke or other cerebrovascular disease, substance use, and tuberculosis.

Variant predominance was defined using national trends based on the date in which >50% of sequenced cases were identified from a given variant [20]. Omicron was the predominant variant from 25 December, 2021 onwards.

### 2.3. Statistical Analysis

We estimated the peak antibody response and decay rate following infection and assessed whether these varied by participant characteristics. Peak antibody concentrations were estimated from measurements taken 30–45 days after illness onset. Participants without an antibody measurement within this timeframe were excluded from the analysis of peak concentrations. Participants with at least two samples taken 30 or more days after infection onset were eligible for the analysis of antibody decay rates. If an immunological event expected to boost antibody response occurred during follow-up, that individual’s antibody trajectory was censored from the time of the event onwards. Censoring events included (1) receipt of a COVID-19 vaccine dose or a new SARS-CoV-2 infection documented in their medical record and (2) a more than two-fold increase in antibody concentration more than 45 days after illness onset for a given target antigen (presumed undocumented infection or vaccination).

Participant-level peak antibody concentrations and decay rates were estimated using a Bayesian approach. Peak antibody concentrations were assumed to follow a log-normal distribution. Antibodies were assumed to follow a log-linear decay trajectory. Both the peak antibody concentration and decay rate were assumed to be functions of individual-level covariates, with the decay rate including a participant-level random slope term. Estimated peak antibody concentrations and decay rates from the fitted models were used to calculate the expected antibody concentration one year post-peak and the expected time to seroreversion. Baseline antibody levels were not modeled.

Model parameters were estimated via Markov Chain Monte Carlo (MCMC) using the rstan package. Convergence was assessed using the Gelman-Rubin statistic (R-hat). All point estimates reported are the median of the posterior distribution. Differences between groups were assessed using the 95% credible intervals derived from the posterior distribution of the difference between the relevant group-level parameter estimates. All analyses were conducted using R version 4.1.3. Detailed model specifications can be found in the Supplementary Materials.

As monoclonal antibodies were available for use during the study period and affect antibody levels, the impact of the receipt of monoclonal antibodies for treatment during the acute illness was explored. Because monoclonal antibodies were only approved for use in adults during the study period and were predominately used in the outpatient setting, we performed a supplemental analysis while adjusting for monoclonal antibody use among the subset of participants 18 years and older who were not hospitalized.

A descriptive analysis of boosting during follow-up was also conducted among adults and children with documented receipt of a COVID-19 vaccine dose or positive SARS-CoV-2 test during follow-up. Samples were not eligible for inclusion in the boosting analysis if they were collected in the first 45 days after the participant’s acute illness or if both vaccination and reinfection occurred between the two sample collection dates. The proportion of samples with a two-fold or greater rise in antibody concentration was estimated by taking the ratio of the antibody concentration from the last pre-event sample and the first post-event sample. Overall, 149 participants were vaccinated, and 47 were reinfected during follow-up, of whom 85 vaccinated and 16 reinfected participants were eligible for the analysis.

## 3. Results

This analysis included 230 participants (194 adults and 36 children, 67% of 342 enrolled participants) with a laboratory-confirmed SARS-CoV-2 infection (181 [78.7%] tested with a PCR test, 39 [17.0%] with an antigen test, and 10 [4.3%] with an unknown test type); 190 (165 adults and 25 children) were included in the peak concentration analysis, and 186 (163 adults and 23 children) were included in the decay rate analysis. The characteristics of the participants are provided in Supplementary Table S1. Among the adults, 75% were female, 84% had completed a primary vaccination series, and 38% had evidence of prior infection at the time of illness onset. Among the children, 47% were female, 50% were vaccinated, and 17% showed evidence of prior infection. Most participants were symptomatic (97%) and presented for care in an outpatient setting (89%). The participants in the decay analysis provided 984 unique measurements each for anti-S and anti-N antibodies. Supplementary Figure S1 shows the antibody trajectories for all participants.

**Figure 1:**
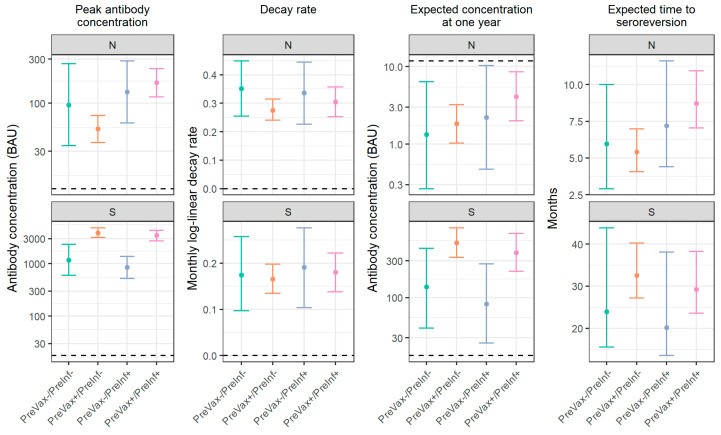
Results of antibody decay model based on prior immunological exposure status prior to enrollment. Median is used as point estimate. Error bars give 95% credible interval. For peak antibody concentration and expected concentration at one year, dashed lines show cutoff for seropositivity. For decay rate, dotted line is at zero, indicating no decay. Note: immunological exposure groups were defined according to participant’s history of vaccination and infection prior to their current SARS-CoV-2 infection. Based on self-report (prior infection and vaccination), medical record review (prior vaccination), and baseline anti-nucleocapsid seropositivity (restricted to samples collected within 1 week of illness onset), participants were classified as either having history of both vaccination and infection (PreVax+/PreInf+), history of vaccination but not infection (PreVax+/PreInf−), history of infection but not vaccination (PreVax−/PreInf+), or history of neither vaccination nor infection (PreVax−/PreInf−) at baseline. Sample sizes for immunologic exposure groups are PreVax+/PreInf+ion (n = 80), PreVax−/PreInf+ (n = 15), PreVax+/PreInf− (n = 80), and PreVax−/PreInf− (n = 11).

Key differences were observed in the antibody response based on prior immunological exposure status (Figure 1; Supplementary Table S2; and Supplementary Figure S2). The median peak anti-N concentrations were the lowest among the participants with a prior history of vaccination but not infection (53 BAU; 95% credible interval [CI]: 37–73). Compared to this group, the peak anti-N concentrations were significantly higher for the participants with prior vaccination and infection (167 BAU; 95% CI: 117–237) and participants with prior infection but no prior history of vaccination (132 BAU; 95% CI: 61–286). No significant difference was found for the participants with history of neither prior infection nor vaccination. In contrast, compared to the participants with no history of vaccination (with or without a history of infection), the peak anti-S antibody concentrations were significantly higher among the participants with a history of vaccination but not infection (3861 BAU; 95% CI: 3132–4802) and participants with a history of vaccination and infection (3441 BAU; 95% CI: 2725–4318).

Based on our models, the anti-N antibody levels in all groups were estimated to be below the level of detection (seronegative) by one year, while anti-S antibodies were estimated to remain detectable (seropositive). For the anti-N antibodies, the estimated time to seroreversion following infection ranged from 5.4 months (95% CI: 4.1–7.0) among participants with a prior history of vaccination but not infection to 8.7 months (95% CI: 7.0–10.9) among participants with a prior history of vaccination and infection, with significant differences only being observed between the participants with a history of vaccination with and without a history of prior infection. For the anti-S antibodies, the median time to seroreversion ranged from 20 to 33 months, with no significant differences between groups. There were no significant differences in decay rates for the anti-N or anti-S antibodies between immunological exposure groups.

The associations observed between immunological exposure and peak antibody concentrations and decay rates remained after adjusting for age, sex, comorbidities, hospitalization status, and SARS-CoV-2 variant (Table 1). Participants infected with the Omicron variant had significantly higher peak anti-N antibody concentrations than those infected with pre-Omicron variants (relative concentration: 2.53; 95% CI: 1.58–4.05), although we were unable to adjust for the number of exposures. Children aged 0–12 had significantly lower peak anti-S concentrations compared to adults aged 18–49 (relative concentration: 0.06; 95% CI: 0.04–0.11). The decay rate for anti-S antibodies was higher in males compared to females (difference in decay rate: 0.06; 95% CI: 0.00–0.11).

Among adult outpatients, participants who received monoclonal antibodies had significantly higher peak anti-S antibody concentrations (relative concentration: 1.85; 95% CI: 1.37–2.50) and lower decay rates (difference in decay rate: −0.09; 95% CI: −0.16–−0.09). The relationships between other participant characteristics and anti-S peak concentrations and decay rates were not significantly impacted by adjustment for monoclonal antibody use (Supplementary Figure S3). No significant associations were found between monoclonal antibody use and anti-N peak concentrations and decay rates.

Over the 12 months of follow-up, 149 participants were vaccinated (of which 133 were previously vaccinated and received an additional dose and 16 received their first dose), and 47 were reinfected (defined by having a documented positive COVID-19 test). Among the participants with eligible pre- and post-event samples, a two-fold or greater boost in the anti-S antibody concentration was observed in 22% (19/85) of those vaccinated during the follow-up and 31% (5/16) of those reinfected during the follow-up. A two-fold or greater increase in anti-N titers was observed for 81% (13/16) of those who were reinfected during the follow-up. The average time between the event and post-event sample was 68 days for vaccination events and 70 days for reinfection events.

## 4. Discussion

This study highlights several key aspects of antibody responses in the year after acute infection in a cohort of Indigenous individuals from communities in the Southwest US with a high burden of disease and a high uptake of COVID-19 vaccines. History of infection and vaccination prior to the acute illness were observed to be significant drivers of the post-infection antibody responses, with differences manifesting primarily for peak antibody levels but not for decay rates. Notably, a prior history of vaccination was associated with higher peak anti-S antibody concentrations following acute infection compared to unvaccinated individuals, while those with a prior history of vaccination but not infection tended to have lower anti-N concentrations than those with a prior history of infection.

In this study, elevated antibody levels were maintained for up to 2 years longer and were more influenced by prior immunologic exposure for anti-S than for anti-N antibodies. Consistent with other studies, the peak anti-S concentrations were higher for vaccinated individuals [21,22] regardless of infection history, but the decay rates were similar across immunologic exposure groups. The estimated median time to seroreversion for anti-S antibodies was also similar across groups with estimates from 20 to 33 months, a range consistent with other studies [23]. While immune response and protection are multi-factorial, anti-S antibody levels have been shown to correlate with neutralizing antibody titers and protection from reinfection [24,25]; thus, these findings support a benefit from vaccination.

In contrast, the anti-N antibody levels dropped below seropositive levels within one year post-peak, with no significant differences between prior immunologic exposure groups. The peak antibody concentrations and resulting time to seroreversion were the highest for those with a prior history of vaccination and infection and the lowest for those with a prior history of vaccination but not infection. This is consistent with prior findings showing a blunted anti-N response to infection in people who have been vaccinated and a faster time to seroreversion for anti-N compared to anti-S antibodies [21,22,23], likely resulting from vaccine-induced immune imprinting against the S protein, leading to decreased dissemination of the virus and partial inhibition of the immune response to the N protein [21,22].

Other individual characteristics were explored, with differences observed based on age and sex. Children younger than 12 years had significantly lower peak anti-S antibody concentrations than adults. This is consistent with other studies that found lower antibody concentrations and reduced functional antibody responses in children compared to adults [26]. In this cohort, factors that may have led to lower antibody concentrations in children than adults include more asymptomatic and non-severe illness, fewer comorbidities that increase the risk of prolonged infection, and fewer children completing their primary vaccination series compared to adults. Compared to adults, children also have a more robust innate immune response and lower levels of expression of angiotensin-converting enzyme 2 (ACE2), the receptor that SARS-CoV-2 uses for host entry [27,28]. In this study, faster anti-S antibody decay rates were observed in men. This was observed in prior studies and is likely driven by a multifactorial set of genetic and biologic factors which give rise to sex-mediated differences in immune function [29,30,31].

This study had several limitations. This study enrolled a small cohort of individuals with predominantly symptomatic acute infection who did not require hospitalization. The results therefore may not be fully generalizable to the overall population of these two communities in the Southwest or other Indigenous populations. Small sample sizes for certain groups, particularly hospitalized individuals and children, may have limited our ability to make inferences. There is also the potential for misclassification, especially for history of infection due to waning of anti-N antibodies, baseline anti-N antibodies potentially reflecting an early antibody response to the current infection, and the occurrence of subclinical infections. The proportion of participants exhibiting a greater than two-fold rise in titers after vaccination or reinfection during the follow-up is likely underestimated due to waning that occurs between the boost and the time of the next sample collection. Finally, this study focused on IgG antibodies; however, IgA and IgM antibodies also play important roles in the immune response to SARS-CoV-2 infection [32,33] and may have different drivers.

Indigenous persons are underrepresented in COVID-19 research [14,15], with few studies conducted on the drivers of immunological responses to SARS-CoV-2 vaccination or infection in Indigenous populations [34,35]. This study addressed this gap in the United States and found that history of infection and vaccination prior to the acute illness were significant drivers of the post-infection antibody responses, providing support for the benefits of vaccination. This study provides valuable information to understand the immunological landscape in a population that experienced a high burden of disease and had a high uptake of COVID-19 vaccines [36], which may inform both continued SARS-CoV-2 control efforts and responses to future epidemics.

## Figures and Tables

**Table 1:** Results from analysis of peak antibody concentrations and decay rates.

	Difference in Monthly Decay Rate			
	Nucleocapsid		Spike	
	Unadjusted	Adjusted **	Unadjusted	Adjusted **
**Age group (in years)**				
0 to 12	−0.02 (−0.19, 0.15)	−0.04 (−0.21, 0.14)	0.09 (−0.05, 0.23)	0.06 (−0.08, 0.21)
13 to 17	0.04 (−0.06, 0.14)	0.05 (−0.06, 0.15)	0.04 (−0.04, 0.12)	0.04 (−0.04, 0.13)
18 to 49	REF	REF	REF	REF
50 to 64	0.05 (−0.01, 0.12)	0.07 (−0.01, 0.14)	0.02 (−0.03, 0.08)	0.01 (−0.05, 0.07)
≥65	0.02 (−0.07, 0.11)	0.04 (−0.06, 0.13)	−0.03 (−0.10, 0.05)	−0.01 (−0.09, 0.06)
**Male sex**	0.03 (0.03, 0.10)	0.01 (−0.06, 0.08)	0.06 (0.01, 0.11) *	0.06 (0.01, 0.11) *
**Prior immunological status**				
PreVax−/PreInf−	REF	REF	REF	REF
PreVax+/PreInf−	−0.07 (−0.18, 0.03)	−0.07 (−0.18, 0.04)	−0.01 (−0.10, 0.08)	0.02 (−0.06, 0.11)
PreVax−/PreInf+	−0.02 (−0.16, 0.13)	0.00 (−0.16, 0.15)	0.02 (−0.10, 0.14)	0.04 (−0.08, 0.15)
PreVax+/PreInf+	−0.05 (−0.16, 0.06)	−0.03 (−0.15, 0.06)	0.01 (−0.09, 0.09)	0.05 (−0.05, 0.14)
**Inpatient hospitalization**	0.01 (−0.07, 0.10)	0.01 (−0.10, 0.09)	0.06 (−0.01, 0.13)	0.07 (0.00, 0.14)
**Any comorbidities †**	−0.02 (−0.08, 0.04)	−0.02 (−0.08, 0.05)	−0.01 (−0.06, 0.04)	−0.01 (−0.06, 0.04)
**Omicron variant**	−0.03 (−0.09, 0.02)	−0.04 (−0.10, 0.02)	−0.02 (−0.06, 0.03)	−0.01 (−0.06, 0.03)
	**Relative peak antibody concentration**			
**Age group (in years)**				
0 to 12	1.42 (0.49, 4.22)	2.41 (0.85, 7.01)	0.08 (0.04, 0.15) *	0.06 (0.04, 0.11) *
13 to 17	0.62 (0.23, 1.55)	0.57 (0.23, 1.39)	0.77 (0.44, 1.32)	0.71 (0.45, 1.12)
18 to 49	REF	REF	REF	REF
50 to 64	0.67 (0.38, 1.19)	0.64 (0.37, 1.10)	1.15 (0.82, 1.59)	1.08 (0.80, 1.44)
≥65	0.57 (0.28, 1.23)	0.55 (0.27, 1.09)	1.21 (0.79, 1.86)	1.21 (0.85, 1.77)
**Male sex**	1.24 (0.75, 2.06)	1.34 (0.81, 2.19)	0.74 (0.52, 1.05)	0.98 (0.75, 1.28)
**Prior immunological status**				
PreVax−/PreInf−	REF	REF	REF	REF
PreVax+/PreInf−	0.55 (0.19, 1.71)	0.45 (0.16, 1.28)	3.34 (1.59, 6.81) *	4.22 (2.40, 7.61) *
PreVax−/PreInf+	1.37 (0.38, 5.04)	1.41 (0.42, 4.83)	0.73 (0.31, 1.66)	0.86 (0.43, 1.67)
PreVax+/PreInf+	1.74 (0.60, 5.15)	1.29 (0.46, 3.88)	2.94 (1.41, 6.05) *	3.10 (1.71, 5.58) *
**Inpatient hospitalization**	0.89 (0.43, 1.84)	0.75 (0.36, 1.49)	0.55 (0.34, 0.90) *	0.76 (0.52, 1.10)
**Any comorbidities †**	0.89 (0.53, 1.50)	0.95 (0.58, 1.60)	1.34 (0.95, 1.89)	0.94 (0.73, 1.23)
**Omicron variant**	2.53 (1.58, 4.05) *	2.55 (1.57, 4.12) *	1.19 (0.85, 1.63)	0.86 (0.67, 1.11)

REF: reference group. * Significant at the 95% credible level. ** The adjusted estimates are from a model including age, sex, immunological status, hospitalization status, presence of any comorbidity, and variant. † Includes cancer, chronic kidney disease, chronic liver disease, chronic lung disease, COPD, asthma, cystic fibrosis, dementia or other neurologic conditions, diabetes, disabilities, heart conditions, hypertension, HIV, immunocompromisation, mental health conditions, overweight and obesity, pregnancy, sickle cell disease, thalassemia, being a current or former smoker, receipt of organ or stem cell transplant, stroke or other cerebrovascular disease, substance use, and tuberculosis. Immunological exposure groups were defined according to the participant’s history of vaccination and infection prior to their current SARS-CoV-2 infection. Based on the self-report (prior infection and vaccination), medical record review (prior vaccination), and baseline anti-nucleocapsid seropositivity (restricted to samples collected within 1 week of illness onset), the participants were classified as either having a history of both vaccination and infection (PreVax+/PreInf+), a history of vaccination but not infection (PreVax+/PreInf−), a history of infection but not vaccination (PreVax−/PreInf+), or history of neither vaccination nor infection (PreVax−/PreInf−) at baseline.

## Data Availability

Tribal regulations prohibit unauthorized data sharing. Persons wishing to access deidentified study data for purposes concordant with the IRB-approved protocol and informed consent forms may submit a request to the corresponding author for review by the appropriate tribal data oversight entity.

## Supplementary Materials

The following supporting information can be downloaded at https://www.mdpi.com/article/10.3390/v16101573/s1, Figure S1: Observed antibody trajectories, Figure S2: Observed antibody trajectories by immunological exposure group; Figure S3: Relative peak antibody concentration and difference in decay rates for anti-S antibodies among adult outpatient participants; Table S1: Sociodemographic characteristics of participants at enrollment by age group, Table S2: Summary antibody concentrations (BAU) by immunologic status; Supplemental Methods.
