# Supplementary material for: The Trajectory of Antibody Responses One Year Following SARS-CoV-2 Infection among Indigenous Individuals in the Southwest United States
Source: Viruses. 2024 Oct 5;16(10):1573. doi: 10.3390/v16101573 (PMC11512241; doi:10.3390/v16101573)
Supplement: Supplementary file 1 [file viruses-16-01573-s001.zip › viruses-3175927-Supplementary Materials.pdf]

Supplementary Material

The trajectory of antibody responses one year following SARS-CoV-2 infection among Indigenous individuals in the Southwest United States

Claire P. Smith, Rachel M. Hartman, Alexa M. Kugler, Verlena Little, Owen R. Baker, Tarayn A. Fairlie, Rey Fernandez, Melissa B. Hagen, Elvira Honie, Oliver Laeyendecker, Claire Midgley, Dennie Parker, Marqia Sandoval, Saki Takahashi, Laura L. Hammitt, Catherine G. Sutcliffe

Supplementary Tables

Table S1. Sociodemographic characteristics of participants at enrollment by age group

Table S2. Summary antibody concentrations (BAU) by immunologic status

Supplementary Figures

Figure S1. Observed antibody trajectories

Figure S2. Observed antibody trajectories by immunological exposure group

Figure S3. Relative peak antibody concentration and difference in decay rates for anti-S antibodies among adult outpatients

Supplementary Methods

Table S1. Sociodemographic characteristics of participants at enrollment by age group

	Adults (n=194) n (%)	Children (n=36) n (%)
**Median age in years (IQR)**	46.3 (35.3-59.2)	12.2 (5.5-14.7)
**Age group (in years)**		
<5	-	9 (25.0)
5 to 17	-	27 (75.0)
18 to 49	111 (57.2)	-
50 to 64	57 (29.4)	-
≥65	26 (13.4)	-
**Female sex**	145 (74.7)	17 (47.2)
**Enrollment site (service unit)**		
Chinle	130 (67.0)	30 (83.3)
Tuba City	28 (14.4)	3 (8.3)
Whiteriver	36 (18.6)	3 (8.3)
**Running water in home**[a]	158 (81.4)	30 (83.3)
**Wood used to heat home**	122 (62.9)	22 (61.1)
**Education level (of mother for children)**[a]		
Some high school or less	20 (10.3)	3 (8.3)
High school diploma or GED	59 (30.4)	12 (33.3)
Some college or AA	86 (44.3)	9 (25.0)
Completed degree, including graduate	22 (11.3)	7 (19.4)
Missing or prefer not to answer	7 (3.6)	5 (13.9)
**Medical condition or risk factor present**		
Any	148 (76.3)	15 (41.7)
Alcohol and/or substance abuse	15 (7.7)	NA
Asthma	28 (14.4)	5 (13.9)
Cancer	6 (3.1)	0 (.)
Chronic lung disease	1 (0.5)	0 (.)
Current or former smoker	32 (16.8)	NA
Chronic kidney disease	4 (2.1)	0 (.)
Chronic liver disease	9 (4.6)	0 (.)
Diabetes (type I or II)	61 (31.4)	0 (.)
Disabilities[b]	2 (1.0)	4 (11.1)
Heart condition, excluding hypertension	13 (6.7)	0 (.)
Hypertension	50 (25.8)	1 (2.8)
Immunocompromised[c]	3 (1.5)	0 (.)
Mental health condition	45 (23.2)	0 (.)
Obesity	57 (29.4)	8 (22.2)
Pregnancy	2 (1.0)	0 (.)
Supplemental oxygen use at home	3 (1.6)	0 (.)
**Vaccination status at time of acute illness**		
Unvaccinated	26 (13.4)	6 (16.7)
Partially vaccinated	6 (3.1)	12 (33.3)
Completed primary series[d]	162 (83.5)	18 (50.0)
Median time (days) since last dose (IQR)	222 (124-299)	149 (76-209)
**Medical presentation for acute illness**		
Outpatient[e]	172 (88.7)	33 (91.7)
Inpatient	22 (11.3)	3 (8.3)
**Of hospitalized, severe**[f]	0 (.)	0 (.)
**Symptomatic acute illness**	191 (98.5)	32 (88.9)
**Received monoclonal antibodies for acute illness**	75 (38.7)	0 (.)
**Received antivirals for acute illness**	43 (22.2)	0 (.)
**Self-reported or serologic evidence of prior infection**[a,g]		
No	103 (53.1)	19 (52.8)

Yes	73 (37.6)	6 (16.7)
**Variant predominance for acute illness[h]**		
Pre-Omicron	70 (36.1)	17 (47.2)
During Omicron and sub-variants	124 (63.9)	19 (52.8)

AA, Associate of Arts degree; GED, General Educational Development; IQR, interquartile range.

[a] 12 (5.2%) participants missing education level; 2 (0.9%) missing running water in home; 29 (12.6%) missing self-reported or serologic evidence of prior infection

[b] Disabilities includes cerebral palsy, down syndrome, and developmental delays

[c] 2 adults were immunocompromised due to chemotherapy, and 1 adult was immunocompromised due to immunosuppressive medication

[d] Completed primary series = Received at least two doses of an approved mRNA COVID-19 vaccine primary series or one dose of an approved non-mRNA vaccine ≥14 days prior to illness onset. May or may not have received ≥1 booster dose. Of the 162 adults with completed primary series, 107 received the Pfizer-BioNTech vaccine, 44 received the Moderna vaccine, 3 received a mix of the Pfizer-BioNTech and Moderna vaccines, 5 received one dose of the Janssen vaccine, and 2 received the Janssen vaccine followed by an mRNA vaccine. Of the 18 children with completed primary series, all received the Pfizer-BioNTech vaccine.

[e] Outpatient = participants enrolled at outpatient clinics, Emergency Departments, or SARS-CoV-2 testing clinics

[f] Severe COVID-19-associated hospitalization was defined among patients with COVID-19-associated hospitalization according to the World Health Organization's definition and included at least one of the following clinical outcomes occurring during the course of illness: use of supplemental oxygen by non-invasive ventilation or high-flow nasal cannula; intubation or mechanical ventilation; use of vasopressors; dialysis; extracorporeal membrane oxygenation (ECMO); or death.

[g] Serologic evidence of prior infection determined by blood specimen positive for nucleocapsid IgG antibody. Only blood specimens collected within 1 week of illness onset included (total n=134; adults n=115; children n=19).

[h] Variant predominance was defined as the period during which Omicron was detected in >50% of sequenced cases using national trends (Lambrou AS, et al. MMWR 2022). Omicron was the predominant variant from December 25, 2021 onwards.

Table S2. Summary antibody concentrations (BAU) by immunological exposure status.

	Nucelocapsid							
	PreVax-/PreInf-		PreVax+/PreInf-		PreVax-/PreInf+		PreVax+/PreInf+	
	Median (IQR)	Sample size	Median (IQR)	Sample size	Median (IQR)	Sample size	Median (IQR)	Sample size
Baseline:	1.2 (0.6-3.0)	11	1.1 (0.5-2.3)	85	30 (12-314)	14	42 (17-420)	86
Peak (1m):	79 (68-139)	9	64 (26-128)	87	179 (68-372)	16	150 (54-794)	78
6 month:	21 (6.5-39)	8	14 (9.3-24)	46	47 (25-105)	8	29 (12-78)	50
12 month:	5.3 (4.1-6.5)	2	8.6 (5.4-18)	9	60 (45-65)	3	8.1 (5.0-10)	5
	**Spike**							
	PreVax-/PreInf-		PreVax+/PreInf-		PreVax-/PreInf+		PreVax+/PreInf+	
	Median (IQR)	Sample size	Median (IQR)	Sample size	Median (IQR)	Sample size	Median (IQR)	Sample size
Baseline:	48 (4.3-4139)	11	2568 (989-5934)	85	497 (10-2526)	14	2882 (1251-4745)	86
Peak (1m):	1500 (244-5207)	9	5120 (4532-6323)	87	1881 (360-2239)	16	4947 (2485-5629)	78
6 month:	164 (54-735)	8	3077 (1251-4570)	46	564 (190-1407)	8	1753 (703-3067)	50
12 month:	811 (408-1214)	2	794 (449-1732)	9	256 (169-445)	3	840 (613-957)	5

IQR: interquartile range

Note: Immunological exposure groups were defined according to the participant's history of vaccination and infection prior to their current SARS-CoV-2 infection. Based on self-report (prior infection and vaccination), medical record review (prior vaccination), and baseline anti-nucleocapsid seropositivity (restricted to samples collected within 1 week of illness onset), participants were classified as either having a history of both vaccination and infection (PreVax+/PreInf+), history of vaccination but not infection (PreVax+/PreInf-), history of infection but not vaccination (PreVax-/PreInf+), or history of neither vaccination nor infection (PreVax-/PreInf-) at baseline.

Figure S1: Observed antibody trajectories.

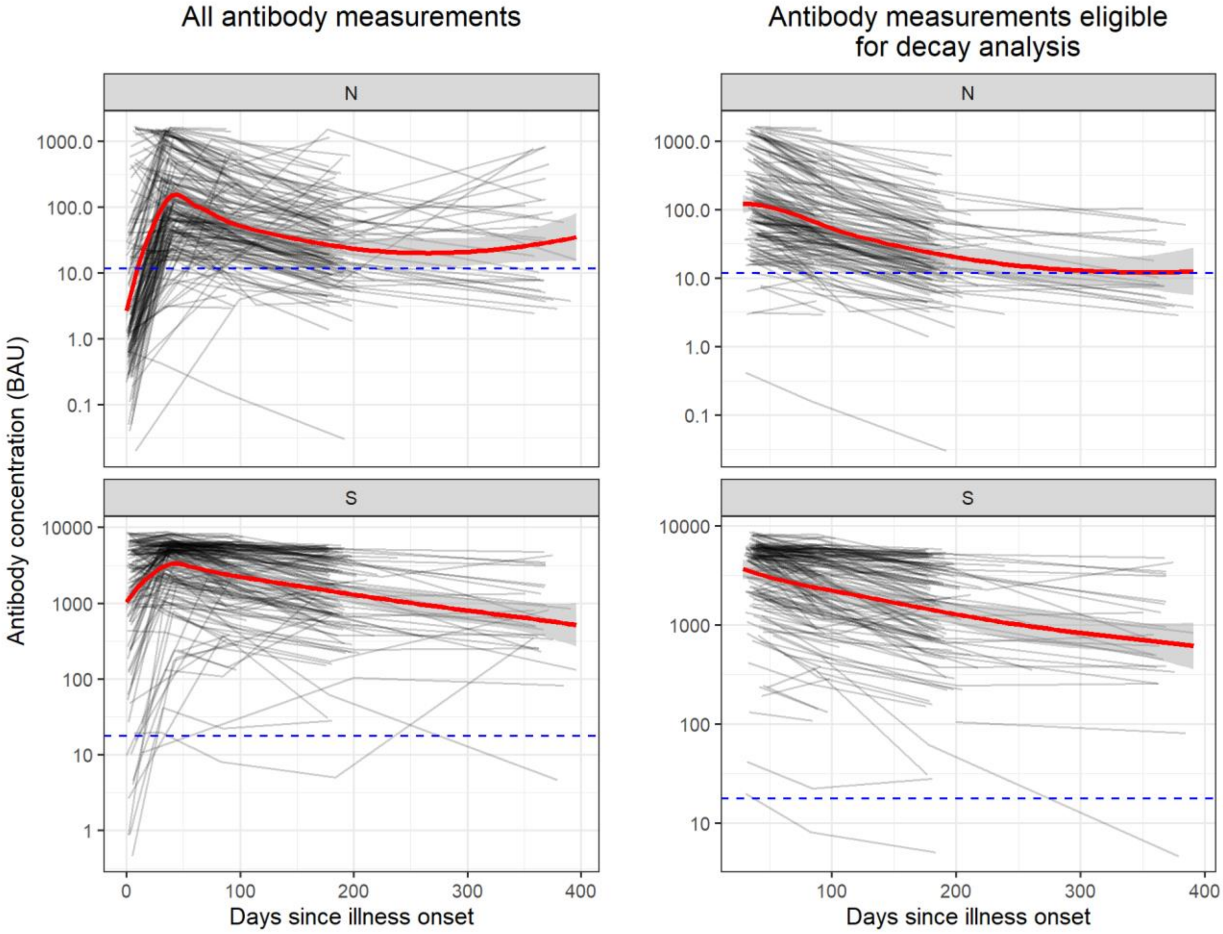

The dashed lines show the cutoff for seropositivity. The red line gives the locally estimated scatterplot smoothing (LOESS) fit for the data.

Figure S2: Observed antibody trajectories by immunological exposure group.

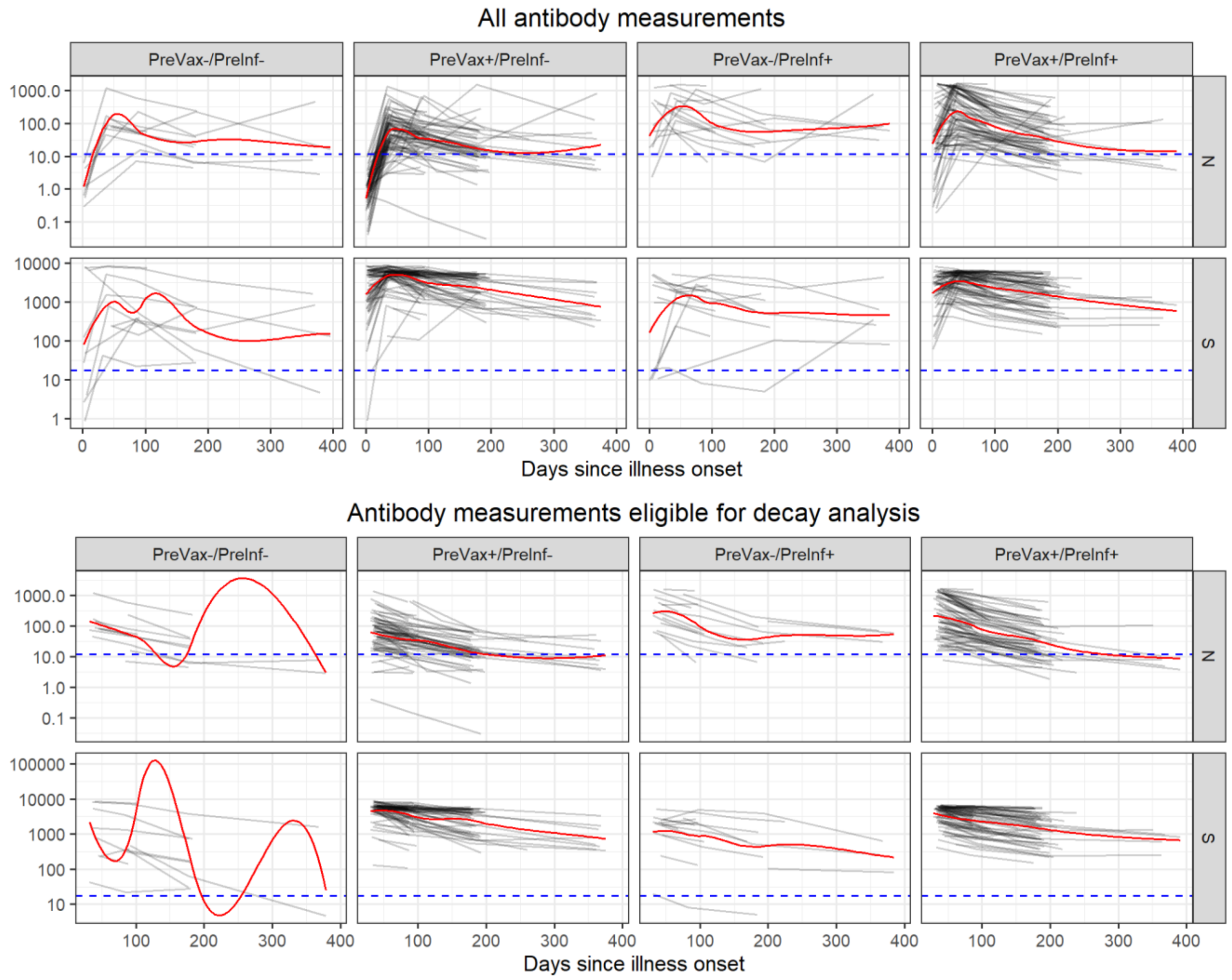

The dashed lines show the cutoff for seropositivity. The red line gives the LOESS fit for the data.

Note: Immunological exposure groups were defined according to the participant's history of vaccination and infection prior to their current SARS-CoV-2 infection. Based on self-report (prior infection and vaccination), medical record review (prior vaccination), and baseline anti-nucleocapsid seropositivity (restricted to samples collected within 1 week of illness onset), participants were classified as either having a history of both vaccination and infection (PreVax+/PreInf+), history of vaccination but not infection (PreVax+/PreInf-), history of infection but not vaccination (PreVax-/PreInf+), or history of neither vaccination nor infection (PreVax-/PreInf-) at baseline.

Figure S3: Relative peak antibody concentration and difference in decay rates for anti-S antibodies among adult outpatient participants.

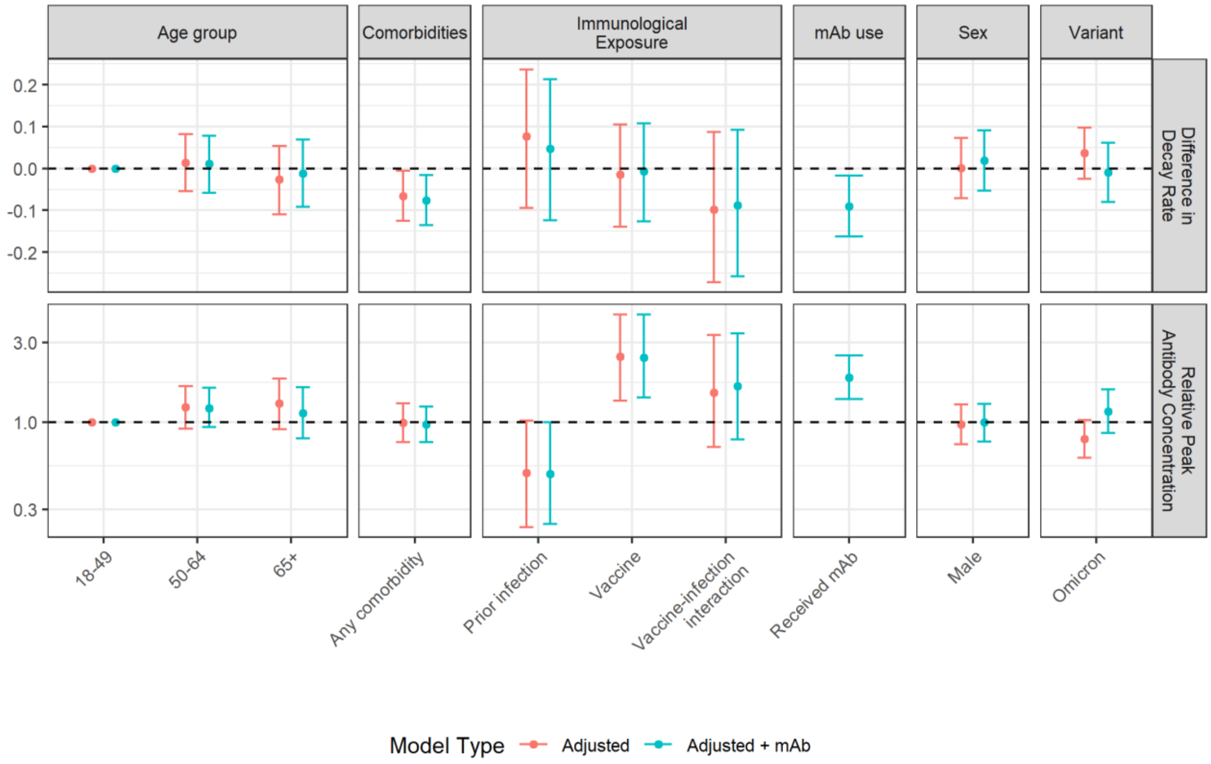

The red model is adjusted for immunological exposure group, age, comorbidity, sex, and variant. The blue model is adjusted for immunological exposure group, age, comorbidity, sex, variant, and use of monoclonal antibodies. The median is used as the point estimates. Error bars give the 95% credible interval.

Supplementary Methods.

Let X_i denote the matrix of covariates for participant i. We modelled a participant's peak antibody concentration, P_i, as being log-normally distributed:

$$\log(P_i) \sim N(\beta_0 + \beta X_i, \ \sigma_p^2)$$

We assumed that antibody concentrations decayed in a log-linear fashion over time. Let A_{i,t_1} and A_{i,t_2} be two sequential antibody measurements from participant i taken at times t_1 and t_2. Under the log-linear decay model,

$$\log\left(A_{i,t_1}\right) - \log\left(A_{i,t_2}\right) \sim N((\alpha_0 + \alpha X_i + \gamma_i)(t_2 - t_1), \ \ \sigma_d^2)$$

Where γ_i is an individual-level random slope term drawn from a normal distribution with mean zero and variance σ_γ^2.
